# Scandinavian perspectives on life support at the border of viability

**DOI:** 10.3389/fped.2024.1394077

**Published:** 2024-04-24

**Authors:** Janicke Syltern

**Affiliations:** ^1^ Department of Neonatology, St. Olavs Hospital University Hospital, Trondheim, Norway; ^2^ Department of Clinical and Molecular Medicine, Faculty of Medicine and Health Sciences, Norwegian University of Science and Technology, Trondheim, Norway

**Keywords:** neonatology, extreme premature infants, ethical decision-making, life support, perinatal palliative care

## Abstract

Advances in neonatal medicine have allowed us to rescue extremely preterm infants. However, both long-term vulnerability and the burden of treatment in the neonatal period increase with decreasing gestational age. This raises questions about the justification of life support when a baby is born at the border of viability, and has led to a so-called “grey zone”, where many professionals are unsure whether provision of life support is in the child's best interest. Despite cultural, political and economic similarities, the Scandinavian countries differ in their approach to periviable infants, as seen in their respective national guidelines and practices. In Sweden, guidelines and practice are more rescue-focused at the lower end of the border of viability, Danish guidelines emphasizes the need to involve parental views in the decision-making process, whereas Norway appears to be somewhere in between. In this paper, I will give an overview of national consensus documents and practices in Norway, Sweden and Denmark, and reflect on the ethical justification for the different approaches.

## 1 Introduction

The Scandinavian countries share a common social democratic political tradition, were solidarity and equality has been core values. In line with this, the health systems incorporate tax-based funding, publicly owned and operated hospitals, universal access based on residency, and comprehensive coverage. Scandinavian countries are among those with the lowest child mortality rate in the world, and survival rates for preterm infants are also among the highest in the world. In a joint publication of survival rates for extremely premature infants in all Nordic countries born in 2021, the overall survival among live born infants was 58% at 22–24 weeks and 91% at 25–27 weeks gestation (1). There was no statistically significant differences in survival rates among live born and admitted infants between regions and countries. Live born infants at 25–27 and at 28–31 weeks gestation were almost universally admitted for neonatal care, in all regions. However, differences were found in admission rates for infants born alive at 22–24 weeks of gestation: 73% in Denmark, 92% in Norway and 98% in Sweden (1). The authors discuss that the lower rates in Denmark most likely reflects variations in attitudes and different guidelines on perinatal management at the border of viability.

Periviable birth is defined as delivery occurring from 20 0/7 weeks to 25 6/7 weeks gestation (2). Most infants born at or above 26 weeks gestation with access to a modern NICU will survive, and infants born before 22 weeks are virtually nonviable. The most important prognostic factor is gestational age, but there also are other factors, both non-modifiable (growth, plurality, infection, fetal sex) and modifiable (antenatal steroids and immediate access to NICU level 3) that will affect prognosis. Gestational age-based thresholds for periviable resuscitation have been criticized, both due to the uncertainty in the estimate of gestational age (±7 days), the disregard of these other prognostic factors and the “Cinderella-effect”, with a sudden change in approach at the stroke of midnight (3). The Scandinavian guidelines include individualized approach to ameliorate this aspect, but as we will see, the thresholds differ.

There are also differences in the involvement of parents in the decision-making process. In case of periviable birth, the parents are those who will live with the consequences: taking the surviving infant home, or mourn a death. Currently, a majority of obstetric and neonatology organizations recommend shared decision-making with the pregnant person and family as the child's surrogate. In a recent commentary, high-profile international neonatologists, ethicists and parent advocates urges that individualized decision-making with families should take precedence over any mandatory policies (4).

How are these trends reflected in the different national consensus documents and practice trends in the Scandinavian countries? I will start by presenting the documents in chronological order, focusing on both gestational age limits and attitudes towards parental involvement.

## 2 Scandinavian guidelines

### 2.1 Norway: the 1998 consensus statement and practice trends

The Norwegian consensus statement for treatment for extremely preterm infants was issued more than 25 years ago. The Research Council of Norway organized a national consensus conference in order to establish medical and ethical sound thresholds for initiation of life support, inviting a multidisciplinary group including neonatologists from different NICUs, psychologists, nurses and ethicists (5). Discussions centered on challenges of predicting sequelae and the burdens of treatment for both the infant and the family, legal and ethical issues and international practice variation. They concluded that decisions should be based on ethical deliberation that involved all affected parties, guided by “*the infant's illness and life prospect*”, but added that “*consideration for the family must be given weight*” (5). Before 23 weeks gestation, life support should be seen as futile and be considered experimental. Treatment at 23 and 24 weeks should be optional and based on the infant's vitality and the individual physician's judgement. Parents' opinions should be given weight, but there should be no doubt that the physician carried the responsibility for the final decision. From 25 weeks, life support should be considered standard of care, unless there were other major negative prognostic markers (5). Since 1998, neonatologists in Norway seem to have become more willing to provide life support at lower gestational ages than recommended by the 1998 guideline. The mean reported gestational age threshold among Norwegian neonatologists for resuscitating infants had decreased by almost a week from 23^+6^ in 1998 to 23^+0^ weeks in 2005 (6).

Due to concerns about varying approaches to the provision of life support when faced with premature delivery between 22 and 25 weeks gestation among Norwegian hospitals, the National Council for Priority Setting in Health Care conducted a questionnaire study in 2015 on current practices and local guidelines (7). They found two different approaches among the eight Norwegian NICUs that treated infants below 26 weeks: (a) Units mainly providing life support to all infants from 23^+0^ weeks gestation and (b) Units providing life support to all infants from 24^+0^ weeks, making an individual judgment of infants born at 23 weeks. These findings were also reflected in data from the Norwegian Neonatal Network: in the period 2009–2014, the proportion of live-born infants transferred to a NICU was nearly 97% at 24 weeks, 74% at 23 weeks and 19% at 22 weeks (8). During those years, only eight infants born at 22 weeks were transferred to NICU care. In conclusion, the grey-zone in Norway appeared to have moved from 24 to 23 weeks; at 22 weeks life support was still rarely offered, and at 24 weeks, life support was normally started.

The level of parental involvement in decision-making is largely unknown. Most units stated that parental wishes were given decisive weight before 24 weeks gestation, but little is known about how they are involved. Concerns were raised about whether “*parents receive neutral information about prognosis*” (7). The National Council recommended the Norwegian Directorate of Health to start a process with the aim to harmonize practice. So far, this work has not been initiated.

### 2.2 The Swedish 2016 consensus: “proactive care”

The Swedish National Board of Health and Welfare organized in 1989 a national conference for neonatologists and obstetricians, discussing the perinatal management of extremely premature infants. They did not reach agreement about whether extremely premature infant in the range of 23–26 weeks gestation would benefit from initial intensive care, and further studies were suggested (9). During the following decades, significant regional differences in initial approach and survival rates evolved, as became evident in a national, population-based study of extremely premature infants born between 1985 and 1999, and confirmed in the prospective cohort study EXPRESS (The Extremely Premature in Sweden Study), performed between 2004 and 2007 (10, 11).

Increasing survival rates at 22 and 23 weeks gestation in centers providing active care led up to a new consensus between Swedish obstetrician and neonatologists. The 2016 guideline recommends that life support should be considered from 22^+0^ weeks, and recommended from 23^+0^ weeks. This is justified in the guideline by the following statement (traduced from Swedish by the author):

“*an infant born at week 22 + 0 has the right to health care on the same terms as all other people in Sweden. A child born close to the limit of viability has an acute, life-threatening condition that often can be treated successfully. It is impossible to make an accurate prognosis immediately after birth regarding the chance of survival and the risk of future impairments. The default setting should therefore be an active approach unless it is completely obvious that treatment efforts are hopeless*” (12).

Parents should receive information, and the neonatologist and the obstetrician should, “as far as possible, take into account the views of the parents” (12). There are still differing opinions among neonatologists, ranging from a duty-based ethical standpoint that every premature infant should be offered life support (including at 21 weeks) regardless of parental wishes, to standpoints that infants born below 24 weeks should only be given life support if the parents demand so (9).

### 2.3 Denmark: “family-centered approach” still present in the 2018 guideline

Denmark is known for its family-oriented approach, with roots in a popular movement that challenged the technological approach to delivery, birth and neonatal intensive care already during the 1970s. A column published in a national newspaper in 1986 by a pair of parents also contributed to form the public opinion. They told the story of their premature daughter who “lived in a plastic box and died 4 ½ months later” and claimed that “society should not have put all these resources on “before-lives” (“før-liv”):

“*Either we must take the decision-making out of the hands of physicians and say: After this or that limit we do not go further. Or the hospitals, with the physicians in front, must mount to their moral responsibility, not only for the single life, but for all those lives that are affected by what they have created*” (13).

In 1990, a national consensus conference was held with experts informing a lay panel, and the latter recommended not to offer life support below 25 or 26 weeks gestation. The process of decision-making with parents and support to families were central issues. Over the last decade, there has been a trend towards centralization of threatened premature labour at 22 and 23 weeks to enable better informed choice by parents and improved quality of care. These plans did not elicit much criticism or public discussion, suggesting that the public opinion might have changed (13). In 2018, The Danish obstetrician and neonatologist societies agreed on new consensus guidelines: a palliative approach is recommended at 22 weeks, at 23 weeks life support should be individualized and based on gestational age, birth weight, vitality and parental views. Life support should be considered standard of care from 24 weeks of gestation. Conversations with parents should aim at supporting them “*in their decision-making process/preparation for the process at the birth of an immature child. As far as possible, the doctor must reach agreement with the parents on a treatment plan and a treatment level*” (14). However, the clinicians will always assess each infant individually, and prenatal agreements may need to be adjusted, and ongoing discussions with the parents around treatment level during stabilisation and subsequent treatment are warranted.

## 3 Ethical considerations

Both the Norwegian 1998 and the Swedish 2016 consensus statements established that the primary concern in the decision of whether to provide life support at the border of viability was the *best interest* of the child. In the ethical concept of best interest, the principles of beneficence (clinicians' obligation to pursue the infants' good) and nonmaleficence (our obligation to avoid harm) are merged together. The goal is to choose the treatment option where benefits outweighs harms, in order to minimize both undertreatment and overtreatment of extremely premature infants (15). Departing from the same principle, they arrive at different thresholds: the Swedish guidelines stands out by offering intensive care at 22 weeks, and recommending active treatment from 23 weeks gestation. What does it mean for an infant born at 22–23 weeks to pursue survival, both in terms of burdens of treatment on short and long term for the infant, and what are the consequences for parents and siblings?

### 3.1 Survival

Thanks to national clinical quality registries for preterm infants in the Scandinavian countries (the Swedish Neonatal Quality Register, the Danish Newborn Quality Database and the Norwegian Neonatal Network), we have access to updated, population-based survival data. The Swedish and Danish annual reports includes survival of live births at different gestational ages (Table 1). The most recent Norwegian study was published in 2017, examining 1-year survival (Table 1) and rates of major neonatal morbidities among infants born at gestational age 22–26 weeks in 2013–2014 (NEPS 2) (16). Table 1 shows that at 24 weeks, survival in recent years is approximately 70% in all three countries, while there is a wider range for 22–23 weeks, consistent with the different recommendations given in the national consensus documents.

**Table 1:** Survival of extreme premature infants in Scandinavia.

Gestational age	Sweden 2018–22 *N* (%) of live born to discharge	Denmark 2019–2022 *N* (%) of live born to PMA 43 + 6	Norway 2019–2022 % of admitted to discharge	Norway 2013–14 *N* (%) of live born to 1 year
22 weeks	48/119 (40)	xx/xx (3)	22 weeks (36)	3/17 (18)
23 weeks	120/188 (64)	11/50 (22)	23 weeks (65)	12/42 (29)
24 weeks	185/261 (71)	67/94 (71)	24 weeks (73)	35/62 (56)
25 weeks	261/303 (86)	95/127 (75)	25 weeks (85)	59/70 (84)
26 weeks	329/362 (91)	135/153 (88)	26 weeks (88)	76/84 (90)

Swedish Neonatal Quality Register, the Danish Newborn Quality Database and the Norwegian Neonatal Network, NEPS 2 (16–18).

### 3.2 Prognosis for survivors

How is it possible to abstain from life support if there is around 40–60 chance of survival? The short answer is that neonatal morbidity, burden of treatment and subsequent physical and mental disability is inversely correlated with gestational age. The chance of survival without impairment if born alive, increase from 1.2% at 22 weeks to 52% at 26 weeks (19).

In the first national cohort from Norway (NEPS 1), 75% of surviving infants born at 23–25 weeks gestation presented some disability at the age of 5 years (20). At 11 years, 54% of the surviving extremely premature infants without severe disability had at least one mental health problem, and the odds ratio for autism was 4.3 in the extremely premature cohort as compared with a reference group (21). In the NEPS 2 study, more than half of the survivors experienced major neonatal morbidity (16).

Lundgren et al. found that the increased survival in Sweden lamentably did not show a concomitant reduction in neonatal morbidity (22). Thus, the absolute number of infants born before 24 weeks gestation who suffered from severe neonatal morbidity increased. Long-term follow up shows high rates of both neurodevelopmental disorders (75%) and somatic diagnosis (88%) in children born before 24 weeks gestation during 2007–2018 in Sweden (23). Intellectual disability was found in 4 out of 10, and 1 out of 4 presented autism spectrum disorders. Just over half received habilitation services. Neurodevelopmental disorders became more frequent with age and was present in 82% of the children at 10–13 years of age (23).

### 3.3 Burden of treatment

The total burden of intensive care correlates strongly with immaturity, as reflected in length of hospital stay and the invasiveness of treatments. As an example, the median duration of mechanical ventilation in Sweden for a baby born at 22 weeks gestation was 54 days, as compared to 22 days for a baby born at 24 weeks, and 2 days or less for babies born between 25 and 31 weeks (24). In Norway, babies born at 23 weeks gestation spent an average of 32 days on mechanical ventilation, as compared to 10 days for those born at 26 weeks—if they were ever intubated (Data from NNN, 2016–2019). The length of inpatient care for Swedish infants born at 22–23 weeks gestation during 2016–18 was more than five months (162–164 days) on average, and mean age of discharge home was more than ten months (322–329 days) (24). This must be assumed to have a huge impact on family life for those affected.

An infant on mechanical ventilation is exposed to many painful procedures every day. A systematic review of procedures performed in neonates found that on an average, each neonate was exposed to 7.5–17.3 painful procedures per day during their first 14 days in a NICU (25). Periviable infants experienced a higher number of painful procedures, and pain management was more inconsistent in these vulnerable neonates. Research has shown that painful stimuli reach the immature brain already by 20–24 weeks gestation, while regulatory mechanisms do not mature until beyond term. This means that the premature baby is especially vulnerable both to suffer from pain during procedures, and to the long-term damaging effects of pain on brain development (26).

### 3.4 The ethics of perinatal palliative care and shared decision-making

When faced with comparable outcomes and burdens of treatment as presented for an infant born at 22 weeks, autonomous adults are allowed to decline life support and opt for palliative care. The periviable infant cannot raise his or her voice against the invasive treatment. To assume that rescue is paramount therefore means excluding the most vulnerable from the palliative paradigm of care that is open to autonomous adults. In our fight against perinatal mortality, we risk turning the periviable infant into a “mere recipient of technology” (27).

How, and by whom should these decisions be made—by clinicians, or by those closest to the infant, the parents? Bioethicists have argued that when opinions among health care personnel vary, parents should be allowed to make the decision, referring to this as the “zone of parental discretion” (28). The Danish guidelines emphasizes the need to involve parental views in the decision-making process. In Norway, as in other countries, worries about late feelings of guilt are used as arguments against letting parents participate actively in the decision. Such decisions are high-stakes and emotionally fraught. However, the argument that parents need to be protected from the decisional burden to avoid harm has not received empirical support when a model of *shared decision-making* is applied. Higher levels of shared decision-making have been associated with lower grief scores and lower decisional conflict compared to paternalistic or informed decision-making (29). We have suggested one possible approach of shared decision-making where parents are invited into the decision-making space as equal partners, and given support, time and space for reflection (30).

An interesting finding is that clinicians working in neonatal care seem less eager to rescue their own, hypothetical periviable infant when asked in questionnaire studies (31). Whereas 9 out of 10 Norwegian pediatricians would provide life support to an infant born at 24 weeks, only 6 out of 10 would want treatment for their own infant (32). We found that none of the Norwegian pediatric residents surveyed wanted life support for their own hypothetical infant at 23 weeks, and most were negative or not sure at 24 weeks (33).

Whether, or how much, the interests of others may weigh in when it comes to life and death decisions for infants is a controversial issue. In Denmark, the focus on the misery and hardship on both child and family leads to an ethics of responsibility, where the family is seen as the core unit, and parents are supported both as decision-makers and recipients of care (34). This contrast with the Swedish guideline where a periviable infant's right to life support is established without consideration of the family's wishes and preferences.

The parents of an infant born at 22 weeks must see their baby go through numerous painful procedures during months in the NICU. In case of survival, he or her faces a 49% risk of intellectual disability and 28% risk of autism as they grow older (23). This will severely influence not only the child, but also the lives of his or her parents and siblings. As a counterweight to Swedish neonatologists, the Swedish philosopher Tännsjö argues that it can be moral to allow, and even encourage parents to let go of their periviable infant and opt for a new pregnancy with, hopefully, a healthy “replacement” child (35).

## 4 Conclusion

Improved survival rates motivates neonatologists to resuscitate more immature infants. This trend is also seen in the Scandinavian countries, but significant differences is seen within Scandinavia with Sweden promoting the most active approach, with active resuscitation from 22 weeks. Should Norway and Denmark follow the trend of pursuing increased survival in the most immature, or should the Swedes move towards more “responsible” neonatal care, paying more attention to the suffering of the most vulnerable infants and their families? There are weighty moral reasons to involve parents in decisions about life support when the child's best interest is not clear, and the consequences for the family are huge. National guidelines on challenging ethical medical dilemmas ought to reflect society's moral norms, and should be developed by relevant stakeholders, not only medical professionals. There is therefore good reasons to revise the Scandinavian guidelines, in order to promote transparent, consistent, documented, published and clearly communicated decision-making processes that can accurately meet the dilemmas that modern technology entails.
